# Supplementary material for: Predicting fall risk using multiple mechanics-based metrics for a planar biped model
Source: PLoS One. 2023 Mar 27;18(3):e0283466. doi: 10.1371/journal.pone.0283466 (PMC10042378; doi:10.1371/journal.pone.0283466)
Supplement: S1 File — File containing the data used in this work. (ZIP) [file pone.0283466.s001.zip › fallRiskData/Data Overview.docx]

**Markov chain metrics**

The Markov chain metrics are located in markovChainMetrics.mat. Within that file, there is a struct, markovChainMetrics, with the following structure:

markovChainMetrics.<speed>.<double support type>.<perturbation time>.<metric class>.<metric statistics>

where

<speed> is v08, v09, v10, v11, or v12 where the number is speed in m/s times 10. For example, v08 means a speed of 0.8 m/s.

<double support type> is SS (instantaneous double support) or DS (finite time double support)

<perturbation time> is DS00 (perturbation happens at the start of double support), SS00 (perturbation happens at the start of single support), or DS05 (perturbation happens in the middle of single support)

<metric class> is speed, length (step length), time (step duration), XCOM (extrapolated center of mass), FRI (foot rotation indicator), q (joint angle variability), dq (joint velocity variability), LEShort (short-term Lyapunov exponents), LELong (long-term Lyapunov exponents), MFPT (mean first passage time), MFPT_ln (natural log of mean first passage time)

<metric statistics> is mu (normalized mean), var (normalized standard deviation), mu_raw (raw mean), or var_raw (raw standard deviation)

**Brute force metrics**

The brute force metrics are located in bruteForceParams.mat. Within that file, there is a struct, bruteForceParams, with the following structure:

bruteForceParams.<speed>.<double support type>.<perturbation time>.<number steps>.<metric class>.<metric statistics>

where

<speed> is v08, v09, v10, v11, or v12 where the number is speed in m/s times 10. For example, v08 means a speed of 0.8 m/s.

<double support type> is SS (instantaneous double support) or DS (finite time double support)

<perturbation time> is DS00 (perturbation happens at the start of double support), SS00 (perturbation happens at the start of single support), or DS05 (perturbation happens in the middle of single support)

<number steps> is n10, n20, n30, n40, n50, n60, n70, n80, n90, n100, n200, n300, n500, or n1000 where the number indicates the number of steps in the simulation

<metric class> is speed, length (step length), time (step duration), XCOM (extrapolated center of mass), FRI (foot rotation indicator), q (joint angle variability), dq (joint velocity variability), LEShort (short-term Lyapunov exponents), LELong (long-term Lyapunov exponents)

<metric statistics> is mu_vec (mean for each simulation, normalized), mu (normalized mean of means, normalized), var_vec (standard deviation for each simulation, normalized), var (mean of the standard deviations, normalized), mu_vec_raw (raw mean for each simulation), or var_vec_raw (raw standard deviation for each simulation)

**PCA Coefficient**

The mappings from the normalized individual gait parameters to the PCA scores are contained in pcaCoefficients.mat. The variable coeff_LElong contains the mapping using all gait parameters except the short-term Lyapunov exponents. The variable coeff_LEno contains the mapping without any Lyapunov exponents. Each column corresponds to a principle component, in descending order of component variance. Each row corresponds to a gait parameter as given in the table below. To visualize the most significant terms in the mapping, PCA.xlsx contains the mappings, with darker cell colors corresponding to larger values.

| **Number** | **coeff_LElong** | **coeff_LEno** |
| --- | --- | --- |
| 1 | speed mu | speed mu |
| 2 | speed var | speed var |
| 3 | length mu | length mu |
| 4 | length var | length var |
| 5 | time mu | time mu |
| 6 | time var | time var |
| 7 | FRI mu | FRI mu |
| 8 | FRI var | FRI var |
| 9 | XCOM mu | XCOM mu |
| 10 | XCOM var | XCOM var |
| 11 | LElong 1 | q 1 |
| 12 | LElong 2 | q 2 |
| 13 | LElong 3 | q 3 |
| 14 | LElong 4 | q 4 |
| 15 | LElong 5 | q 5 |
| 16 | LElong 6 | q 6 |
| 17 | LElong 7 | q 8 |
| 18 | LElong 8 | dq 1 |
| 19 | LElong 9 | dq 2 |
| 20 | LElong 10 | dq 3 |
| 21 | LElong 11 | dq 4 |
| 22 | LElong 12 | dq 5 |
| 23 | q 1 | dq 6 |
| 24 | q 2 | dq 7 |
| 25 | q 3 | dq 8 |
| 26 | q 4 |  |
| 27 | q 5 |  |
| 28 | q 6 |  |
| 29 | q 8 |  |
| 30 | dq 1 |  |
| 31 | dq 2 |  |
| 32 | dq 3 |  |
| 33 | dq 4 |  |
| 34 | dq 5 |  |
| 35 | dq 6 |  |
| 36 | dq 7 |  |
| 37 | dq 8 |  |
