# Supplementary material for: Predicting fall risk using multiple mechanics-based metrics for a planar biped model
Source: PLoS One. 2023 Mar 27;18(3):e0283466. doi: 10.1371/journal.pone.0283466 (PMC10042378; doi:10.1371/journal.pone.0283466)
Supplement: Attachment — Submitted filename: Response to reviewers.docx [file pone.0283466.s002.docx]

**Response to reviewers of PONE-D-22-21276: Predicting fall risk using multiple mechanics-based metrics for a planar biped model**

The authors thank the reviewers for their informed and detailed assessments of the initial manuscript, especially for the suggestions that the authors feel have significantly improved the paper. Below are the details of the edits made in response to those suggestions. Within the manuscript, changes are in blue.

As we were working through the requested changes, we realized that the results for the combined models used an earlier, incorrect version of the code. We have corrected the results to use the correct code, which changed all of the reported values. The major conclusions of the work did not change.

**Editor Comments:**

This is an interesting paper – thank you for your submission.

Thank you.

Please carefully address the suggestions of reviewer 1.

We have made the changes recommended by reviewer 1. Please see our responses to the reviewer for the details of what we have changed.

Reviewer 2 made some suggestions I would not agree with. E.g. in my opinion it is not necessary to split the introduction into an extra literature review. Also, the suggested additional citations are optional, don’t feel pressured to cite something you feel is not necessary.

Thank you for this guidance. We have made the changes we feel are appropriate and have explained our reasoning when we did not make the suggested change.

**Reviewer 1 Comments:**

This manuscript describes a study in which fall risk is being predicted by metrics calculated from simulations of a model, and from Markov chain simulations. The fall risk is expressed as the MFPT from the Markov chain. Results showed that many strides were needed to get reliable estimates of most measures took many strides, and that single parameters could not predict falls.

Overall, this is interesting work. However, I have the feeling that the authors want too much in this manuscript, which makes everything rather hard to follow. It seems like there are almost 2-3 stories in here; 1. Using Markov chains to calculate gait metrics 2. Effects of length on gait metrics 3. Fall prediction models. By combining these three, I feel that the authors do not really do justice to all of them. In addition, the authors may wish to think about the audience they are writing for. I myself am a biomechanist with (quite) some technical training, but still had quite the hard time following the parts on the Markov chains; I am no expert on this, and details were hard to follow. Hence, I will largely refrain from comments on this. Below are more detailed comments.

Major

1) The models from multiple metrics are nice, but in a way, not very informative. That is, the reader has no clue which metrics are included in the models, and, as such, the models are very hard to judge. It would be good to have at least a table with which metrics load on which PC, and then, one would ideally also like to know how all the PC’s contribute to the model.

As the reviewer points out, a disadvantage of this method is that the model is not easily interpretable.

Very few of the PCA components are closely aligned with any single gait parameter or easily interpreted group of gait parameters. We have added some text to the results section describing this: “In general, the principle components did not closely align with any individual gait parameter or easily interpreted group of parameters. Thus, both models appeared to use information from all of the gait parameters used to create the PCA components.” In the supplemental file, we now include both the PCA mappings and an Excel file to visualize the most significant terms, with darker cell colors corresponding to larger magnitude values. We have also added a new table (Table 6) containing all of the non-zero coefficients in the models.

2) It seems that for the calculation of all parameters, all stride-data is resampled to contain 100 samples. Note that for the Lyapunov exponents, this is not usually done, as usually, the temporal variations in the time series are retained (e.g. resampling 200 strides to 20000 samples, while retaining variations in amount of samples per stride). This could have serious effects on the calculated Lyapunove exponents (and their correlation with mfpt.

The reviewer is correct that this is what was done. In the new “Comparison to Human Gait Parameter Values” results subsection (see response to major comment 3), we have included discussion that most other works do not resample on a per stride basis and that this may change the results: “The Lyapunov exponent values were also substantially different than those found in human experimental studies. The experimental studies typically found short-term Lyapunov exponents on the order of 1 [18, 21, 22] while this study found values on the order of 0.01. The human studies found long-term Lyapunov exponents on the order of 10^‑2^ [18, 21] while this study found mostly negative long-term Lyapunov exponents on the order of 10^-4^. One possibility for this difference is in how the data was processed. In the human studies [18, 21, 22], the entire trial was sampled at a uniform rate. In this study, each step was re-sampled to an equal number of points, which removes temporal information about step duration variability from the data.”

3) Nowhere in the manuscript can the reader find the actual values of any of the outcome parameters, making it very hard to check whether any of the findings make some sense. For instance, for the Lyapunov exponents (see minor comment 3).

We have added in a new table (Table 2) that includes the raw values for one representative case. We have also added in a new “Comparison to Human Gait Parameter Values” results subsection that compares the raw values of the gait parameters to human experimental values. In addition, we added in some discussion of these raw values in the “Markov Chain Metric Validation” results subsection. Finally, the raw values for the brute force method are now included in the supplemental data.

4) As said, I am no expert on Markov chains, but I do find it surprising that so many strides are required to get to the “true” value of the Markov chain simulation. Could this have to do with the fact that in fact, these simulations are different in nature, so that actually, differences are to be expected? Could the authors for instance show that the variance of estimates (or the mean of the estimated) of say mean walking speed, which requires 2000+ strides to be 0.1 unit within the Markov chain simulations, indeed changes over such a long period? I would assume that a simulation of 1000 strides would not lead to a significantly different estimated walking speed from one of say 1500 strides?

The brute force simulation values do appear to be converging to the Markov chain values (see new Fig. 2). We also did this analysis using the longest brute force simulation as the “true” value and found very similar results. Thus, the number of steps to converge does not seem to be because the Markov chain and brute force simulations are fundamentally different, except perhaps for the Lyapunov exponents. This is now stated in the paper: “This indicates that the brute force values are converging to the Markov chain values, and thus, that the two methods are capturing fundamentally the same gait parameters.”

We also added some discussion about why the number of steps required may be so much higher than previously reported: “This difference is partly due to the definition of accurate enough, which is not standardized across studies. This work used normalized values to define accuracy, and if the scaling factor was small, the difference in dimensional values may be insignificant even if the difference in normalized values is relatively large. For example, for mean step duration, a normalized value of 0.5 corresponds to a dimensional value of 0.08 s, which for many practical cases, may be sufficiently accurate.”

Minor

1) Line 88 “without slip”, line 89 “fail by slipping” how is this possible, giving line 88?

In the simulation, we enforced the no slip condition kinematically regardless of the required ground reaction forces. However, if the required coefficient of friction exceeded 0.7, we treated the gait as failed because we assume it would have slipped. We have clarified the language to better describe what we did: “the stance foot was assumed to roll along the ground without slip. ... In addition, a step could fail by requiring a coefficient of friction greater than 0.7…”

2) It is unclear how the brute force simulations were created. The text talks of “initial set of 10 random perturbation vectors”, but the siimulations were for 100 steps. Does this mean not every step is perturbed?

Each vector had a length of 1000. Every step was perturbed. We have clarified this in the text: “To generate these data, an initial set of 10 random perturbation vectors of length 1000 were created. These vectors were then used to simulate each gait with perturbations every step for 1000 steps.”

3) (this could be major?); line 157-158: “Long term Lyapunov exponents are usually 2 orders of magnitude larger than short term Lyapynov exponents”; no, they are not. Actually, they are usually 2 orders of magnitude smaller. A table with actual values of the metrics (see major comment 3) would help here. Edit; I checked the data (would be nice to also have raw values in the bruteforcemetrics, and not only normalized values), and it seems that indeed the short term is an order of magnitude largerd than the short term?

You are correct that our long-term Lyapunov exponents are about 2 orders of magnitude smaller than our short-term Lyapunov exponents. We have corrected this statement in the text.

As requested, we now include the raw values in the supplemental data file.

4) Formula 12 misses a quadratic term for PCA2?

We have added this term in for clarity. We have also modified the description of the equation: “Similar to the individual fall risk models, a quadratic regression was used that initially included many possible linear and quadratic terms but no interaction terms:”

5) Line 302 states that Xpca are the transformed gait metrics, but it would seem that these are instead the PC scores formed from (Several) gait metrics?

Yes, you are correct. We were using transformed gait metrics to mean the PCA scores. We have updated the terminology to make this clear: “PCA scores formed by combining multiple gait metrics.”

6) It seems that the m/2 cannot be right in formula 12? As for m=3 this would lead to beta3 and XPCA,1.5. I think this should be floor(m/2), or some other operation.

We have updated the numbering in this equation to use two number subscripts, with the first number matching the PCA score and the second number indicating if the term is linear or quadratic.

**Reviewer 2 Comments:**

This paper has proposed a predicting model to predict fall risk using multiple mechanics-based metrics for a planar biped model.

However, authors need to clarify the following doubts.

1) In the abstract section, I think it will be wise to talk about directly about Falls in case of Robots only, no need to bring the human factor. Readers might get confused.

We have chosen to leave the discussion about humans in the abstract. Many of the tested metrics have only been considered in the case of human stability. In addition, we have added a new results subsection that compares the values in this work to human values. Thus, we feel it is important to discuss both humans and robots in the abstract.

2) In the abstract section, you mentioned “Many mechanics-based fall risk metrics have been proposed and validated to varying degrees, including the extrapolated centre of mass, the foot rotation index, Lyapunov exponents, joint and spatiotemporal variability, and mean spatiotemporal parameters”.

Is it only about metrics? What is your proposed model that you must emphasis on abstract rather than metrics.??

We apologize, but we are unsure what the reviewer’s confusion is specifically.

3) Introduction and literature survey should be as made separate paragraphs. First , in the introduction section introduce your problem statement with several recent literature and may be few existing available solution you can mentions(as citations). The at the end write 4 to 5 objectives that you have achieved in this work.

3) After introduction, you can have 2. Literature review: where you talk about the available solutions as citations(recent)

That means

 Introduction

 Literature Review.

We have decided to keep the introduction and literature review combined as is typical for biomechanics and robotic papers (and as approved by associate editor).

4) In the materials and methods talk about the materials first then method(proposed one i.e Biped Model). First you write about the materials i.e about the data sets.

We chose to keep the ordering the same because the data sets come from the biped model, so it does not make sense to talk about the data before talking about the biped model.

5) In the method section write Algorithms(proposed).

We have changed the title of subsection “Predicting MFPT” to “Proposed Algorithms for Predicting MFPT.”

6) Figure 2 : Actual vs predicted on test data or train that you mention, there is no point having actual vs predicted for train data.

Figure 3 (formerly Fig. 2) shows the gaits used to create the model. This is now stated in the caption title: “Fall risk model prediction ($\hat{y}$) compared to the true MFPT for the Markov chain data used to create the model.” We included this plot because it shows the best-case results and the amount of error that is present even when using the training data.

7) Which ever following errors you can find, please add them to your work

variance accounted for (VAF),

relative average absolute error (RAAE),

root means absolute error (RMAE),

coefficient of determination (R2),

standard deviation ratio (RSR),

mean absolute percentage error (MAPE),

Nash–Sutcliffe coefficient (NS),

root means squared error (RMSE),

weighted mean absolute percent error (WMAPE),

and mean absolute percentage Error (MAPE,

We now report the adjusted coefficient of determination (training data only), root mean square error, mean absolute error, the mean absolute percentage error, the relative average absolute error, the maximum absolute error, the standard deviation of the error (brute force simulations only), the percent of predictions within tolerance, the three metrics quantifying the fit of the actual and predicted MFPT. We do this for the training data (Table 5), the brute force simulations of the training gaits (Table 7), and the brute force simulations of the validation gaits (Table 8). The equations for metrics that are less commonly used are given in the “Evaluation of Fall Risk Models” section.

8) Clarify the figure 3 also(is it for test set or train set?)

Fig. 4 (formerly Fig. 3) is for both. Subfigure (a) is for the training data and subfigure (b) is for the testing data. The caption now reads: “(a) shows results for the training gaits that were used to create the model, and (b) shows results for the validation gaits that the model had never seen.”

9) Significance of Piped model not written on the manuscript, please talk about the significance of choosing the models(proposed) for the work, in what sense they better than the existing ones. Also do a comparative study with the existing model in other paper.

The authors apologize, but they are unclear what the reviewer means by the “piped model.” We are also unsure what other paper the reviewer is referring to. We have, however, added in some additional motivation for using the combined model “Because the MFPT may be dependent on multiple metrics, two regression models each using multiple metrics were created.”

9) You must cite the following papers in the introduction and literature sections.

While the authors thank the reviewer for suggesting additional papers, we chose not to include these additional references because they do not strongly relate to this work. As this is already a long paper, we wanted to keep the introduction fairly focused.

A) Dinegdae, Y. H., Onifade, I., Jelagin, D., & Birgisson, B. (2015). Mechanics-based top-down fatigue cracking initiation prediction framework for asphalt pavements. Road Materials and Pavement Design, 16(4), 907-927.

B) Roy, S. S., Roy, R., & Balas, V. E. (2018). Estimating heating load in buildings using multivariate adaptive regression splines, extreme learning machine, a hybrid model of MARS and ELM. Renewable and Sustainable Energy Reviews, 82, 4256-4268.

C) Robertson, S. W., & Ritchie, R. O. (2008). A fracture‐mechanics‐based approach to fracture control in biomedical devices manufactured from superelastic Nitinol tube. Journal of Biomedical Materials Research Part B: Applied Biomaterials, 84(1), 26-33.

D) Samui, P., Roy, S. S., & Balas, V. E. (Eds.). (2017). Handbook of neural computation. Academic Press.

E) Mitros, Z., Sadati, S. H., Henry, R., Da Cruz, L., & Bergeles, C. (2022). From theoretical work to clinical translation: Progress in concentric tube robots. Annual Review of Control, Robotics, and Autonomous Systems, 5, 335-359.

F) Roy, S. S., Samui, P., Deo, R., & Ntalampiras, S. (Eds.). (2018). Big data in engineering applications (Vol. 44). Berlin/Heidelberg, Germany: Springer.
