# Supplementary material for: Predicting fall risk using multiple mechanics-based metrics for a planar biped model
Source: PLoS One. 2023 Mar 27;18(3):e0283466. doi: 10.1371/journal.pone.0283466 (PMC10042378; doi:10.1371/journal.pone.0283466)
Supplement: Attachment — Submitted filename: Response to reviewers - rev 2.docx [file pone.0283466.s003.docx]

**Response to reviewers of PONE-D-22-21276R1: Predicting fall risk using multiple mechanics-based metrics for a planar biped model**

Below are the details of the edits made in response to those suggestions. Within the manuscript, changes are in blue.

**Reviewer 1 Comments:**

The authors have improved the manuscript, and I feel it makes a nice contribution to the literature now. I have only one small comment regarding the new figure 2; please indicate (in the figure, and caption) what the 0.10 and 0.5 refer to.

Thank you.

For Fig. 2, we have added the following sentence to the caption to indicate what the 0.10 and 0.50 refer to: The percentage of simulations within 0.1 or 0.5 normalized units of the Markov chain metric values is shown.

**Reviewer 2 Comments:**

The paper is rejected as the authors did not cite the concerned paper and also the gives explanations are not convincing.

As indicated by the associate editor for the previous revision, citing the suggested papers was not required.
